# Changes in sleeping energy metabolism and thermoregulation during menstrual cycle

**DOI:** 10.14814/phy2.14353

**Published:** 2020-01-24

**Authors:** Simeng Zhang, Haruka Osumi, Akiko Uchizawa, Haruka Hamada, Insung Park, Yoko Suzuki, Yoshiaki Tanaka, Asuka Ishihara, Katsuhiko Yajima, Jaehoon Seol, Makoto Satoh, Naomi Omi, Kumpei Tokuyama

**Affiliations:** ^1^ International Institute for Integrative Sleep Medicine (WPI‐IIIS) University of Tsukuba Tsukuba Japan; ^2^ Graduate School of Comprehensive Human Science University of Tsukuba Tsukuba Japan; ^3^ Department of Pharmaceutical Sciences Faculty of Pharmacy and Pharmaceutical Sciences Josai University Sakado Japan

**Keywords:** distal‐to‐proximal skin temperature gradient, energy expenditure, menstrual cycle

## Abstract

Women with ovulatory menstrual cycles show an increase in body temperature in the luteal phase, compared with follicular phase, particularly during the night. Several, albeit not all, studies reported higher energy expenditure in the luteal phase compared with follicular phase. Q_10_ of biological reactions lies between 2.0 and 3.0, predicting a 7‐12% increase in energy expenditure when body temperature rises by 1°C. In this study, temperature dependence of energy expenditure was assessed by comparing changes in sleeping energy expenditure and thermoregulation with menstrual cycle in 9 young females. Energy expenditure was measured using a metabolic chamber, in which sleep was recorded polysomnographically, and core body temperature and skin temperature were continuously monitored. Distal‐to‐proximal skin temperature gradient was assessed as an index of heat dissipation. In the luteal phase, a significant increase in average core body temperature (+0.27°C) and energy expenditure (+6.9%) were observed. Heat dissipation was suppressed during the first 2 hr of sleep in the luteal phase, compared with follicular phase. Rise in basal body temperature in the luteal phase was accompanied by increased energy expenditure and suppressed heat dissipation. The 6.9% increase in metabolic rate would require a Q_10_ of 12.4 to be attributable solely to temperature (+0.27°C), suggesting that energy expenditure in the luteal phase is enhanced through the mechanism, dependent and independent of luteal‐phase rise in body temperature presumably reflects other effects of the sex hormones.

## Graphical abstract

Body temperature, heat production and heat dissipation during sleep was compared between follicular and luteal phase. During the luteal phase, significant increase in average core body temperature (+0.27°C) and energy expenditure (+6.9%) was observed, and temperature dependence of energy expenditure assessed as Q10 was 12.4. Heat dissipation was suppressed during the first 2 hr of sleep in the luteal phase, compared with follicular phase

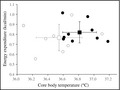

## 1 INTRODUCTION

Women undergo modifications of body temperature during the menstrual cycle. After the postovulatory progesterone rise, core body temperature increases above the follicular phase values (Charloudian & Stachenfeld, 2014; de Mouzon, Testart, Lefevre, Pouly, & Frydman, 1984); higher mesor and lower amplitude of the 24 hr body temperature rhythm, mainly due to elevated nocturnal values in the luteal phase (Shechter, Boudreau, Varin, Diane, & Boivin, 2011). Although this luteal phase rise in basal body temperature has been used as a marker of ovulation for decades (de Mouzon et al., 1984), its relation to heat production and heat dissipation remains inconclusive.

A majority of the studies, with indirect calorimetry over a 24 hr period and/or during sleep, reported higher energy expenditure in the luteal phase compared with follicular phase (Bisdee, James, & Shaw, 1989; Hessemer & Bruck, 1985; Horvath & Drinkwater, 1982; Howe, Rumpler, & Seale, 1993; Meijer, Westerterp, Saris, & Hoor, 1992; Webb, 1986). On the other hand, some studies failed to detect differences in energy expenditure during the daytime (Bisdee et al., 1989; Bittel & Henane, 1975; Frascarolo, Schutz, & Jéquier, 1990; Howe et al., 1993; Stephenson & Kolka, 1985), except one study (Solomon, Kurzer, & Calloway, 1982). Inconsistency in the effect of menstrual cycle on energy metabolism during the daytime is likely due to the effects of meal and physical activity on energy metabolism, the so‐called “masking effect”, which is essentially absent during sleep. Thus, the luteal‐phase rise in core body temperature and energy expenditure was consistently observed during sleep.

Association of body temperature and energy expenditure has been pointed out since the earliest days of indirect calorimetry. From basal metabolic rate of pathological conditions such as typhoid, malaria, erysipelas, etc., DuBois derived that a 1°C increase in temperature is associated with about a 13% increase in metabolic rate (Du Bois, 1921). The increase in energy expenditure at higher body temperature is plausible, since higher temperature stimulates metabolism manifested as Q_10_ effect, a parameter to measure the temperature dependence of a chemical reaction. In majority of the cases, Q_10_ of biological reactions lies between 2.0 and 3.0, the rate of chemical reaction becomes 7‐12% faster when the temperature rises by 1°C (Hochachka & Somero, 1984). It remains unknown, however, whether the increase in body temperature in the luteal phase is plausible to explain the magnitude of the increase in energy expenditure.

With regard to heat loss, distal skin regions are considered the major sites as they are rich in arteriovenous anastomoses which regulate skin blood flow (Bergersen, 1993). Opening of arteriovenous anastomoses selectively increases the flow of warm blood to distal cutaneous vascular beds, which is monitored as distal minus proximal skin temperature gradient (DPG) (Kräuchi, Cajochen, Möri, Hetsch, & Wirz‐Justice, 1997; Kräuchi et al., 2014) and provides a valuable measure for internal heat conduction. An excellent correlation between skin temperature gradient and fingertip blood flow has been reported (Rubinstein & Sessler, 1990). Body heat loss in the evening via an elevated distal skin temperature is the crucial thermoregulatory function for induction of sleepiness and sleep (Kräuchi & Wirz‐Justice, 2001). Decreased skin blood flow and thermal conductance in the luteal phase were observed in one study (Frascarolo et al., 1990) but not in other studies (Kräuchi et al., 2014; Shechter et al., 2011). However, these studies did not focus on heat dissipation when it rapidly changes in the evening.

During sleep, the effect of menstrual cycle on body temperature become larger, and masking effects of meal and physical activity on energy expenditure become smaller. The aim of this study was to assess the impact of menstrual cycle on energy metabolism and thermoregulation during sleep. Energy expenditure and DPG was measured to assess heat production and heat loss, respectively. To examine the plausible causal relation between body temperature and energy metabolism, temperature dependence of energy expenditure was compared with metabolic cost of fever (Du Bois, 1921) and Q_10_ of biological reactions (Hochachka & Somero, 1984).

## 2 METHODS

### 2.1 Subjects

All subjects were recruited by advertisements. Inclusion criteria were as follows: healthy female aged 18–25 years old with a regular menstrual cycle, standard body size (BMI < 25 kg/m^2^), had no subjective sleep complaints, had not been diagnosed with sleep apnea syndrome, no sleeping pills, no shift work or transmeridian travel within 1 month before the study, no exercise habit more than two times a week for the past 6 months, no drinking habit more than three times a week, and no smoking habit. Nine female subjects, who fulfilled inclusion criteria, participated in the study. The study concepts were explained to all the subjects, who provided signed informed consent. The ethics committee of the University of Tsukuba (Ref No., Tai 29–29) approved this study.

### 2.2 Protocol

Sleeping energy metabolism was measured in the follicular and luteal phase, and the two trials were conducted with a randomized repeated‐measures design. Subjects notified the investigators when the menses started, and this was considered day 1 of the menstrual cycle. Measurement was carried out between day 9 to day 13 of their menstrual cycle for the follicular phase (10.4 ± 1.2 days), and day 21 to day 24 for luteal phase (22.7 ± 0.9 days). None of the subjects had a history of taking contraceptive pills.

One week before the experiment, subjects kept a regular sleep/wake schedule of eight hours sleep duration, which was subsequently confirmed by Actigraph recording. Subjects were also instructed to refrain from beverages containing caffeine and alcohol for 3 days before the experiments. To get used to the polysomnographic (PSG) recording system and the whole room indirect calorimetry, the experiment was preceded by an adaptation night in the metabolic chamber a few days in advance.

On the day of the experiment, the subjects ate dinner 5 hr before habitual bed time. After wearing all the sensors and voiding urine, the subjects entered the whole room indirect calorimeter, in which subjects were instructed to maintain a sitting posture until their habitual bed time (23:00‐24:00 hr), and slept for 8 hr.

Urine samples collected during the indirect calorimetry were saved for measuring urinary nitrogen excretion. The body composition was measured using the bioimpedance method (BC‐118E, TANITA Co., Tokyo, Japan) after the subjects exited the whole room indirect calorimeter.

Chronotype and habitual sleep quality of all the subjects were assessed by the Morningness–Eveningness Questionnaire (MEQ) (Horne & Ostberg, 1976) and the Pittsburgh Sleep Quality Index (PSQI) (Buysse, Reynolds, Monk, Berman, & Kupfer, 1989), respectively.

### 2.3 Sleep recording

Sleep was recorded polysomnographically (PSG‐1100, Nihon Kohden, Tokyo, Japan). Six electroencephalogram (EEG) (F3/M2, F4/M1, C3/M2, C4/M1, O1/M2, and O2/M1), two electro‐oculograms, and one submental electromyogram were recorded. Assessments of eye movement by electrooculogram and muscle tone by submental electromyogram were used to identify sleep onset and rapid eye movement sleep (stage R). The records were scored every 30 s to stage N1, stage N2, stage N3, and stage R. Wake after sleep onset was also recorded according to the standard criteria (Berry et al., 2016). Sleep onset latency was determined by the length of time that it took to fall asleep after lights out and classified as stage W. The fast Fourier transform was conducted on the recorded EEG by 5 s to obtain a frequency resolution of 0.2 Hz as previously described (Park et al., 2017). The power content of the delta band (0.75–4.00 Hz) for each 30 s epoch of sleep in μV^2^ was reported.

### 2.4 Energy metabolism

Indirect calorimetry was performed with a room‐size indirect calorimeter (Fuji Medical Science Co., Ltd., Chiba, Japan). The calorimeter room measures the size with 2.00 m × 3.45 m × 2.10 m, having an internal volume of 14.49 m^3^. The chamber is furnished with an adjustable hospital bed, desk, chair, washing basin, and toilet. The air flow in the chamber was ventilated at a rate of 80 L/min. The temperature and relative humidity of the chamber were controlled and maintain with 25.0 ± 0.5°C and 55.0 ± 3.0%, respectively. Concentrations of oxygen (O_2_) and carbon dioxide (CO_2_) in the chamber were measured by online process mass spectrometry (VG Prima δB, Thermo Electron Co., Winsford, UK). Precision of mass spectrometry, defined as the standard deviation for continuous measurement of calibration gas mixture (O_2_ 15%, CO_2_ 5%), was < 0.002% for O_2_ and CO_2_. Hourly average of O_2_ consumption (VO_2_) and CO_2_ production (VCO_2_) rates were calculated using algorithm for improved transient response  (Tokuyama, Ogata, Katayose, & Satoh, 2009).

Energy expenditure and macronutrient oxidation were calculated from VO_2_, VCO_2_, and urinary nitrogen excretion (Ferrannini, 1988). Rate of urinary nitrogen excretion (N), an index of protein catabolism, was assumed to be constant during the calorimetry. Respiratory quotient (RQ) was defined as a ratio of VCO_2_ to VO_2_.

### 2.5 Thermometry

Core body temperature was continuously recorded using an ingestible core body temperature sensor that wirelessly transmits core body temperature to the recorder (CorTemp, HQ Inc, FL, USA). The sensor is accurate to ± 0.1°C, which was calibrated using hot water before use and swallowed 4 hr before bedtime.

Skin temperatures were continuously monitored at eight sites: midforehead, 1 cm above the navel (stomach), right infraclavicular area, midthigh on the right musculus rectus femoris, the center of the middle back of the left and right hand (later averaged) and middle of the left and right foot instep (later averaged). Thermistor probes (ITP082‐24, Nikkiso‐Thermo Co., Tokyo, Japan) connected to data logger (N543, Nikkiso‐Thermo Co.) were fixed to the skin with thin air‐permeable adhesive surgical tape (Transpore Surgical Tape, 3M Science, Tokyo, Japan). Skin temperatures were divided into proximal and distal. The average proximal temperature was calculated by the equation; 0.093 forehead + 0.347 thigh + 0.266 infraclavicular area + 0.294 stomach. The average distal temperature was calculated from the mean of both hands and feet. The difference between distal and proximal skin temperatures was calculated as DPG (Kräuchi et al., 2014).

### 2.6 Q_10_ effect

The temperature dependence of energy expenditure was presented as Q_10_ = (R_2_/R_1_) ^[l0/(T2‐Tl)]^, in which R_1_ and R_2_ were energy expenditure and T1 and T2 were body temperature in the follicular and luteal phase, respectively  (Bennett, 1984).

### 2.7 Assay

Estrogen and progesterone in urine were measured by RIA (LSI Medience Corporation, Tokyo, Japan) and fluorescence enzyme immunoassay (PROG III, Tosoh Bioscience, Inc., San Francisco), respectively. Urinary nitrogen was measured using the Kjeldahl method.

### 2.8 Statistics

Data in the text and figures were given as means ± *SD* of the experimental condition. To compare time course of energy metabolism and body temperature between the follicular and luteal phase, two‐way repeated measures analysis of variance (ANOVA) with post hoc pair‐wise comparisons using the Bonferroni correction was performed. Differences in sleep architecture between the follicular and luteal phase was analyzed using a paired *t*‐test. Statistical analysis was performed using SPSS statistical software (Version 26.0, SPSS Japan, Tokyo, Japan), with the level of statistical significance set at 5%.

## 3 RESULTS

**Figure 1:**
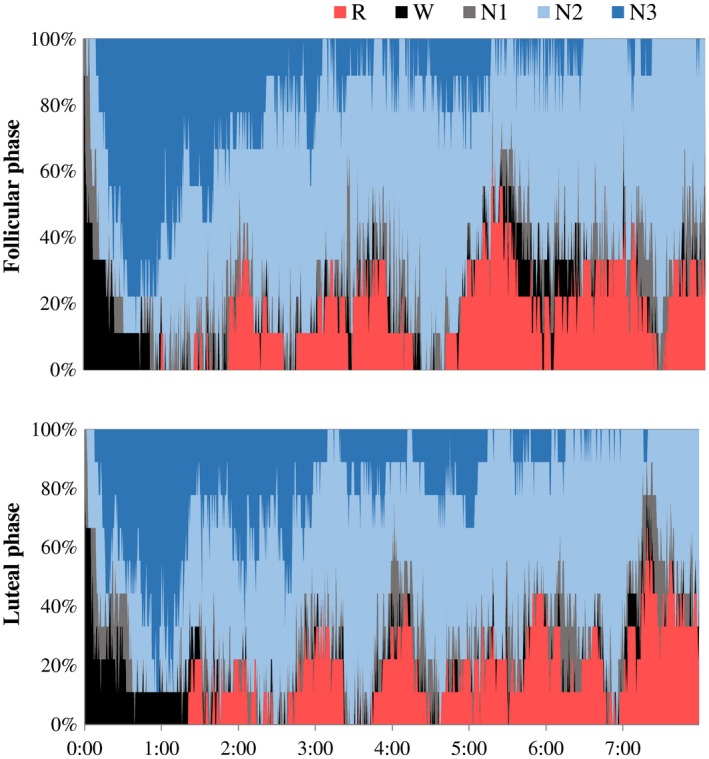
Cumulative display of sleep architecture in nine subjects. The percentage of subjects in each sleep stage was shown; stage W (black), stage N1 (gray), stage N2 (light blue), stage N3 (blue), and stage R (red)

**Figure 2:**
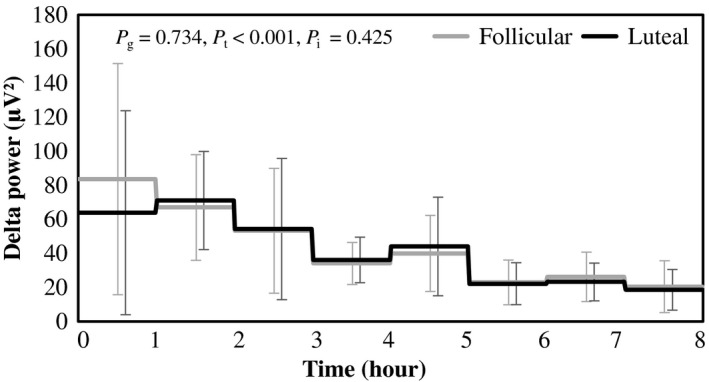
Time course of delta EEG power during sleep in the follicular and luteal phase. Hourly average ± *SD* was shown for follicular (gray) and luteal phase (black). *p* values of two‐way ANOVA were shown as *p*_g_ for the main effect of menstrual cycle, *p*_t_ for the main effect of time and *p*_i_ for the interaction, respectively

Physical characteristics, chronotype and habitual sleep quality of the subjects are shown in Table 1. Urinary excretion of estrogen and progesterone were higher in the luteal phase compared with those in the follicular phase (Table 2). Sleep architecture was comparable between the follicular and luteal phase (Figure 1, Table 3). Delta EEG power, which has been viewed as a measure of intensity of non‐REM sleep, gradually declined during the night (Figure 2). Effect of phase of menstrual cycle and interaction between time and menstrual cycle in delta EEG power were not statistically significant.

**Table 1:** Characteristics of the study population

Age, years	23.3 ± 1.1
Body weight, kg	53.9 ± 9.3
Height, cm	161.8 ± 5.5
MEQ	48.5 ± 5.5
PSQI	5.6 ± 2.3

Values are means ± *SD*.

**Table 2:** Urinary excretion of steroid hormones*

	Follicular	Luteal	*p*‐value
Estrogen, ng/10 hr	6,798 ± 3,748	15,922 ± 6,655	0.001
Progesterone, ng/10 hr	1,277 ± 340	2,127 ± 932	0.015

Note

Values are means ± *SD*.

* Excretion during the 10 hr of indirect calorimetry.

**Table 3:** Sleep architecture

	Follicular	Luteal
Total bedtime, min	480	480
Total sleep time, min	451 ± 27	451 ± 25
Wakefulness, min	19 ± 20	21 ± 24
Sleep latency, min	11 ± 14	8 ± 10
Sleep efficiency, %	95 ± 6	94 ± 5
Stage 1, min	42 ± 28	46 ± 28
Stage 2, min	240 ± 42	230 ± 29
SWS, min	92 ± 27	96 ± 31
REM sleep, min	77 ± 15	79 ± 19
REM sleep latency, min	108 ± 36	108 ± 35
SWS latency, min	26 ± 17	25 ± 17

Note

Values are means ± *SD*.

**Figure 3:**
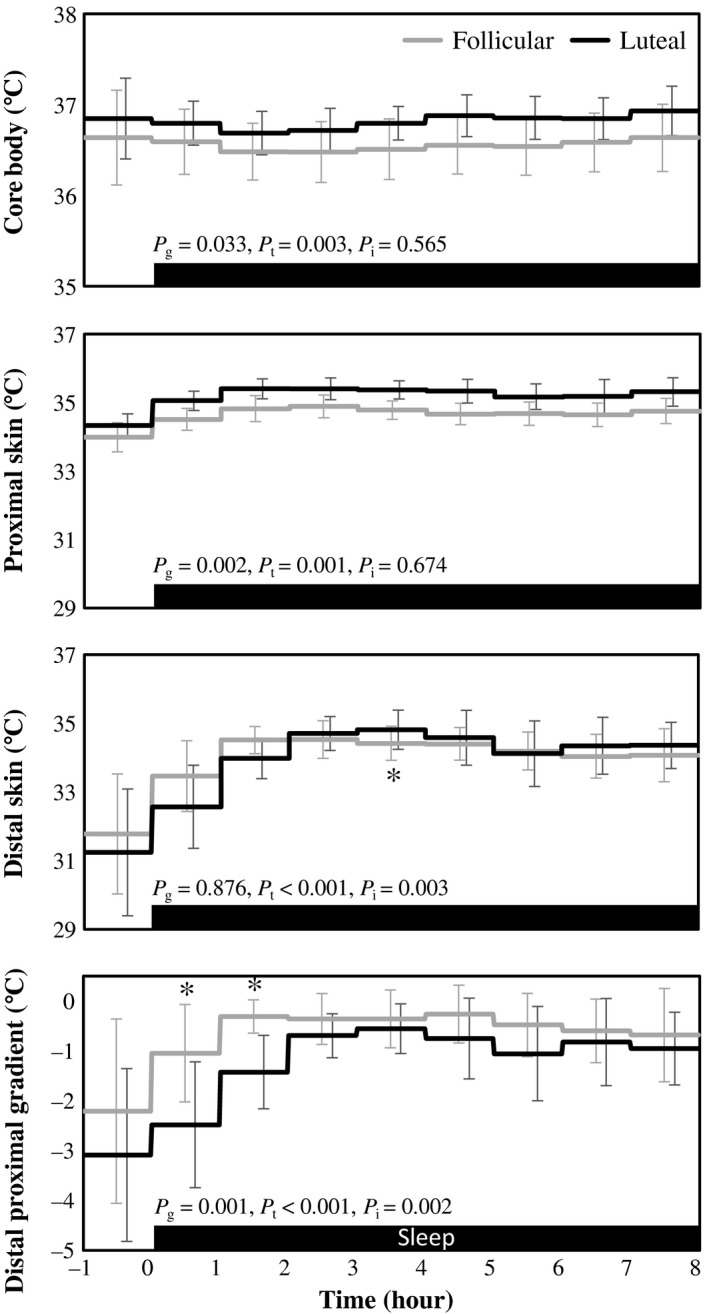
Core body temperature, proximal and distal skin temperature, and distal‐to‐proximal skin temperature gradient. Hourly average ± *SD* was shown for follicular (gray) and luteal phase (black). Gray bar at the bottom indicates sleep period. *p*‐values of two‐way ANOVA were shown as *p*_g_ for the main effect of menstrual cycle, *p*_t_ for the main effect of time and *p*_i_ for the interaction, respectively. *Represents significant difference between follicular and luteal phase by post hoc pair‐wise comparisons using the Bonferroni correction (*p* < .05)

Core body temperature was significantly higher in the luteal phase compared to that in the follicular phase. There was a significant effect of time in core body temperature, but interaction between the time and phase of menstrual cycle was not statistically significant (Figure 3). Proximal skin temperature in the luteal phase was higher than that in the follicular phase, and there was a significant effect of time. Main effect of menstrual cycle on distal skin temperature was not statistically significant, but effect of time and interaction of time and menstrual cycle were significant. During the 4th hour of sleep, distal skin temperature in the luteal phase was significantly higher than that in the follicular phase. Gradient between distal and proximal skin temperature in the luteal phase was larger than that in the follicular phase. It was significantly larger during the first 2 hr of sleep in the luteal phase compared to that in the follicular phase. There was a significant effect of time in DPG; the temperature gradient decreased with the lapse of sleep time.

**Figure 4:**
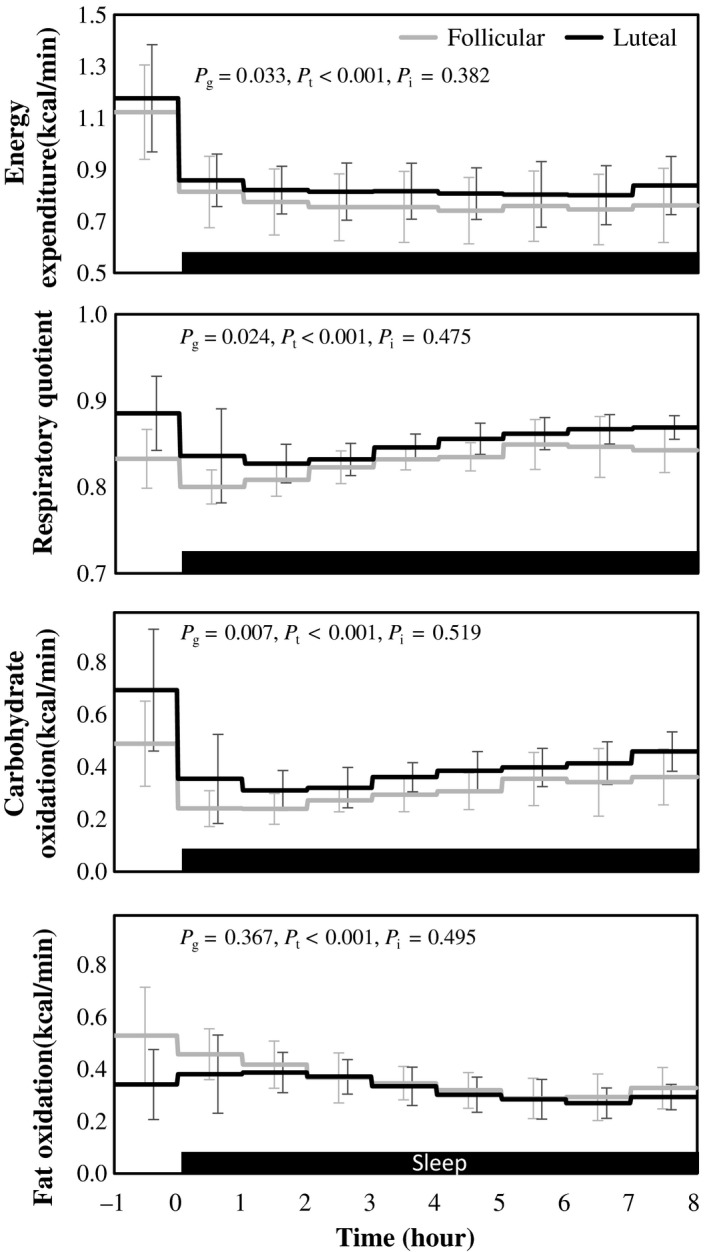
Energy expenditure, respiratory quotient, carbohydrate oxidation, and fat oxidation. Hourly average ± *SD* was shown for follicular (gray) and luteal phase (black). Gray bar at the bottom indicates sleep period. *p*‐values of two‐way ANOVA were shown as *p*_g_ for the main effect of menstrual cycle, *p*_t_ for the main effect of time and *p*_i_ for the interaction, respectively

**Figure 5:**
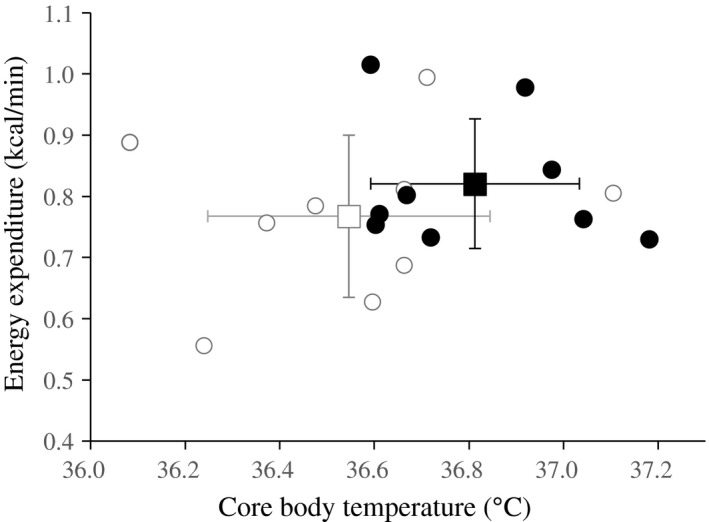
Association between body temperature and energy expenditure during sleep. Averages ± *SD* of 9 subjects was shown for follicular (□) and luteal phase (◼), respectively. Individual data were also presented for follicular(◯) and luteal phase(●), respectively

Energy expenditure showed a significant effect of time and it was significantly higher in the luteal phase compared to that in the follicular phase. Interaction between time and phase of menstrual cycle in energy expenditure was not statistically significant (Figure 4). From the differences in the average body temperature (+0.27°C) and energy expenditure (+6.9%) between the follicular and luteal phase, Q_10_ was estimated as 12.4 (Figure 5).

Respiratory quotient was significantly higher in the luteal phase than that in the follicular phase. Effect of time was significant but interaction between time and phase of menstrual cycle in RQ was not significant. Differences in RQ between the follicular and luteal phase was significantly correlated with differences in urinary excretion of progesterone (*r* ^2^ = 0.416) but not correlated with that of estrogen (*r^2^* = 0.079). Carbohydrate oxidation was significantly higher in the luteal phase than in the follicular phase. Effect of time was significant but interaction between time and phase of menstrual cycle in carbohydrate was not significant. Fat oxidation showed significant effect of time, but the effect of phase of menstrual cycle or interaction between time and phase of menstrual cycle was not statistically significant.

## 4 DISCUSSION

This study confirmed a nocturnal increase in core body temperature in the luteal phase compared with follicular phase. Luteal‐phase rise in core body temperature was accompanied with elevated heat production and suppressed heat loss during sleep.

To address causal relation between the increased energy expenditure and core body temperature in the luteal phase, this study compared the magnitude of the increase in core body temperature and energy expenditure, that is, heat production. An association of body temperature and energy expenditure was reported in 1921 from indirect calorimetry of patients having a fever without shivering such as typhoid, pulmonary tuberculosis and malaria. DuBois derived that a 1°C increase in body temperature is associated with about a 13% increase in metabolic rate (Du Bois, 1921), and attributed the increase in energy expenditure to Q_10_ effect. Compared to the follicular phase, average body temperature (+0.27°C) and energy expenditure during sleep (+6.9%) was significantly higher, and the association between body temperature and metabolic rate (26.0% per °C) was somewhat higher than that reported in patients having a fever (13% per °C). Temperature coefficient (Q_10_) derived from a 0.27°C increase in body temperature and a 6.9% increase in energy expenditure was 12.4, which was higher than the temperature dependency of chemical reaction rate at 2.0‐3.0 (Hochachka & Somero, 1984). The present data indicate that the increase in energy expenditure in the luteal phase is not attributable solely to the increase in body temperature, and energy expenditure is enhanced through the mechanisms, dependent and independent of rise in body temperature in the luteal phase.

Circulating ovarian hormones change across the menstrual cycle, which affect a wide range of physiologic functions. The increase in urinary excretion of estrogen and progesterone in the luteal phase was confirmed in this study. The increase in energy expenditure in the luteal phase has been proposed as a result, at least in part, of an increase in progesterone secretion (Howe et al., 1993; Solomon et al., 1982; Webb, 1986). In studies where increases in energy expenditure were registered, a concomitant increase in progesterone or its metabolic products in urine was observed (Howe et al., 1993; Webb, 1986). Energy expenditure during sleep was correlated with progesterone concentration level in the blood, while there was no significant association between energy expenditure and estradiol (Howe et al., 1993). The use of oral contraceptive agents, a combination of progesterone and estradiol analogue increased the basal metabolic rate (Diffey, Piers, Soares, & O'Dea, 1997). Treatment with depot‐medroxyprogesterone acetate, a potent progestin with a nuclear receptor‐binding affinity of at least twice that of progesterone, increased the resting metabolic rate and body temperature (Steward, Bateman, Slentz, Stanczyk, & Price, 2016).

Heat loss occurs through the entire body surface, but changes in the peripheral skin temperature in the evening are larger than those of the proximal skin temperature. Decrease in core body temperature prior to sleep is accompanied with an increasing distal skin temperature and a reduction in DPG (Cajochen et al., 2005; Kräuchi & Wirz‐Justice, 2001) . Progesterone, which has been shown to be hyperthermic in women (Charkoudian & Stachenfeld, 2016; Charloudian & Stachenfeld, 2014), shifts the onset of the reflex cutaneous vasodilator response to a higher core body temperature, while estrogen may have opposite effects  (Charloudian & Stachenfeld, 2014). Decreased skin blood flow and thermal conductance were observed in the luteal phase (Frascarolo et al., 1990). Consistent with this notion, heat dissipation assessed by DPG in this study was downregulated in the luteal phase, particularly during the first 2 hr of sleep, when nocturnal melatonin secretion occurs. A major component of the nocturnal decline of the core body temperature in males and the follicular phase in females is due to the hypothermic effects of melatonin (Cagnacci, Soldani, & Yen, 1993; Strassman, Qualls, Lisansky, & Peake, 1991). Daytime administration of exogenous melatonin increased peripheral skin temperature and decreased core body temperature of follicular phase female, but the response to melatonin was not observed in the luteal phase (Cagnacci, Soldani, Laughlin, & Yen,^1996^; Cagnacci, Soldani, Romagnolo, & Yen, 1994). The possible mechanism of progesterone to suppress hypothermic effects of melatonin (Cagnacci et al.,^1996^) is consistent with the lack of effect of menstrual cycle on the average of DPG over 24 hr (Kräuchi et al., 2014; Shechter et al., 2011). In addition to suppressing hypothermic effects of melatonin, a direct influence of progesterone on the thermoregulatory center in the hypothalamus (Charloudian & Stachenfeld, 2014) and BAT activity (Quarta, Mazza, Pasquali, & Pagotto, 2012) has been postulated.

It is obvious that an increase in heat production enhances body temperature. The accumulated increase in energy expenditure over 8 hr of sleep in the luteal phase above the follicular phase (+27.4 kcal) was sufficient to raise body temperature by 0.27°C in a 54 kg female (15 kcal). Plausible causal relation among the ovarian steroids, thermoregulation and energy metabolism is as follows; elevated progesterone level suppresses heat dissipation to increase core body temperature, and the increase in core body temperature enhances energy expenditure by a magnitude of which can be explained by Q_10_ effect. In addition, changes in ovarian hormones, probably progesterone, stimulate energy expenditure independent of the luteal phase rise in body temperature. Conversely, the extra heat produced by enhanced energy expenditure further increases core body temperature.

Carbohydrate store is an important determinant of voluntary food intake (Flatt, 1987; Stubbs, Harbron, Murgatroyd, & Prentice, 1995). One previous study (Melanson, Saltzman, Russell, & Roberts, 1996) examined the hypothesis that decreased carbohydrate storage, caused by decreased fat oxidation and increased carbohydrate oxidation, is an underlaying mechanism of luteal phase hyperphagia (Buffenstein, Poppitt, McDevitt, & Prentice, 1995). The previous study, which assessed the resting metabolic rate, did not detect differences in substrate oxidation and RQ. On the other hand, this study could detect an increase in carbohydrate oxidation during sleep in the luteal phase. It is of note that reproducibility of energy expenditure during sleep is higher than that of the resting metabolic rate (Schoffelen & Plasqui, 2018).

Menopause reduced fat oxidation (Santosa & Jensen, 2013), and estrogen therapy over a 12‐month period increased fat oxidation (dos Reis, de Melo, Meirelles, Vezozzo, & Halpern 2003). In animal experiments, carbohydrate oxidation is reduced by estrogen supplementation (Kendrick, Steffen, Rumsey, & Goldberg 1987; Rooney et al., 1993). Thus, the increased RQ during the luteal phase in this study is unlikely due to a higher level of estrogen. Although the effect of progesterone on substrate oxidation has not been well described in the literature, correlated changes in RQ and urinary excretion of progesterone during the menstrual cycle suggests a role of progesterone to shift the oxidized substrate from fat to carbohydrate.

An increase in the circulating level of melatonin (Cain et al., 2010) and heat loss precede sleep onset (Kräuchi, Cajochen, & Wirz‐Justice, 2005). A rapid decline in core body temperature associated with peripheral heat loss increased the likelihood of sleep initiation and facilitates entry into the deeper sleep stage (Campbell & Broughton, 1994; Kräuchi, Cajochen, Werth, & Wirz‐Justice, 1999; Zulley, Wever, & Aschoff, 1981 ), and sleep onset evokes a further decrease in core body temperature (Barrett, Lack, & Morris, 1993). Despite the differences in thermoregulation, sleep architecture was similar between the follicular and luteal phase in the present study. Although some studies have found that rapid eye movement (REM) sleep has an earlier onset, and the percentage of REM sleep tends to decrease in the luteal phase (Baker, Driver, Paiker, Rogers, & Mitchell, 2002; Baker, Driver, Rogers, Paiker, & Mitchell, 1999; Baker et al., 2001; Driver, Dijk, Werth, Biedermann, & Borbély, 1996; Lee, McEnany, & Zaffke, 2000; Lee, Shaver, Giblin, & Woods, 1990; Parry et al., 1999), sleep homeostasis is maintained across the menstrual cycle; percentages of slow wave sleep (SWS), sleep continuity and sleep efficiency remain stable at different phase of menstrual cycling (Baker et al., 2001; Driver et al., 1996; Dzaja et al., 2005; Moline, Broch, Zak, & Gross, 2003). Energy expenditure during sleep is related to sleep stages (Kayaba et al., 2017), and thermoregulatory response is suppressed during REM sleep (Bach, Telliez, & Libert, 2002). In this study, there was no significant difference in sleep architecture between the follicular and luteal phase, suggesting that differences in energy expenditure and thermoregulation between the follicular and luteal phase is not a consequence of the effect of menstrual cycle on sleep architecture.

The following summarizes the key limitations in our study. Dietary intake has a significant impact on energy metabolism. While subjects limited tea, coffee, alcohol, and vigorous exercise 24 hr prior to the measurement, the precalorimetry feeding was not standardized. Cyclical fluctuations in food intake occur in women across the menstrual cycle (Buffenstein et al., 1995). Possibility that hyperphagia stimulates energy expenditure in the luteal phase remained to be evaluated. Causal relations between body temperature and energy expenditure during the daytime may be different from that during sleep. This study assessed energy metabolism and thermoregulation from immediately preceding and throughout the entirety of the sleeping period. To obtain the whole picture of association among heat production, heat dissipation, and core body temperature, evaluation over 24 hr is warranted.

## 5 CONCLUSIONS

Core temperature was found to be elevated by an average of ~ 0.27°C across the night in midluteal relative to the late follicular phase, in conjunction with ~ 6.9% elevation in metabolic rate, but the Q_10_ effect per se was only a minor contributor. Difference in energy expenditure in the follicular and luteal phase presumably reflects other effects of the sex hormones.

## AUTHOR CONTRIBUTIONS

S.Z., H.O., N.O., and K.T. designed the research protocol; S.Z., H.O., A.U., H.H., Y.T., and K.Y. performed indirect calorimetry; Y.S., I.P., and M.S. performed sleep analysis; J.S. performed statistical analysis; S.Z., A.I., and K.T. wrote the paper. The authors declare no conflict of interest.

## References

[phy214353-bib-0001] Bach, V. , Telliez, F. , & Libert, J. P. (2002). The interaction between sleep and thermoregulation in adults and neonates. Sleep Medicine Reviews, 6, 481–492. 10.1053/smrv.2001.0177 12505480

[phy214353-bib-0002] Baker, F. C. , Driver, H. S. , Paiker, J. , Rogers, G. G. , & Mitchell, D. (2002). Acetaminophen does not affect 24‐h body temperature or sleep in the luteal phase of the menstrual cycle. Journal of Applied Physiology, 92, 1684–1691. 10.1152/japplphysiol.00919.2001 11896038

[phy214353-bib-0003] Baker, F. C. , Driver, H. S. , Rogers, G. G. , Paiker, J. , & Mitchell, D. (1999). High nocturnal body temperatures and disturbed sleep in women with primary dysmenorrhea. American Journal of Physiology, 277, E1013–E1021. 10.1152/ajpendo.1999.277.6.E1013 10600789

[phy214353-bib-0004] Baker, F. C. , Waner, J. I. , Vieira, E. F. , Taylor, S. R. , Driver, H. S. , & Mitchell, D. (2001). Sleep and 24 hour body temperatures: A comparison in young men, naturally cycling women and women taking hormonal contraceptives. Journal of Physiology, 530, 565–574. 10.1111/j.1469-7793.2001.0565k.x 11158285 PMC2278431

[phy214353-bib-0005] Barrett, J , Lack, L , Morris, J . (1993). The sleep‐evoked decrease of body temperature. Sleep, 16, 93–99. 8446841

[phy214353-bib-0006] Bennett, A. F. (1984). Thermal dependence of muscle function. American Journal of Physiology, 247, R217–R229. 10.1152/ajpregu.1984.247.2.R217 6380314

[phy214353-bib-0007] Bergersen, T. K. (1993). A search for arteriovenous anastomoses in human skin using ultrasound Doppler. Acta Physiologica Scandinavica, 147, 195–201. 10.1111/j.1748-1716.1993.tb09489.x 8475746

[phy214353-bib-0008] Berry, R. B. , Brooks, R. , Gamaldo, C. E. , Harding, S. M. , Lloyd, R. M. , Marcus, C. L. , et al. for the American Academy of Sleep Medicine. (2016). The AAM Manual for the Scoring of Sleep and Associated Events: Rules, Terminology and Technical Specifications, Version 2.3. Darien, Illinois: American Academy of Sleep Medicine http://www.aasmnet.org

[phy214353-bib-0009] Bisdee, J. T. , James, W. P. , & Shaw, M. A. (1989). Changes in energy expenditure during the menstrual cycle. British Journal of Nutrition, 61, 187–199. 10.1079/BJN19890108 2706224

[phy214353-bib-0010] Bittel, J. , & Henane, R. (1975). Comparison of thermal exchanges in men and women under neutral and hot conditions. Journal of Physiology, 250, 475–489. 10.1113/jphysiol.1975.sp011066 1177148 PMC1348389

[phy214353-bib-0011] Buffenstein, R. , Poppitt, S. D. , McDevitt, R. M. , & Prentice, A. M. (1995). Food intake and the menstrual cycle: A retrospective analysis, with implications for appetite research. Physiology & Behavior, 58, 1067–1077. 10.1016/0031-9384(95)02003-9 8623004

[phy214353-bib-0012] Buysse, D. J. , Reynolds, C. F. 3rd , Monk, T. H. , Berman, S. R. , & Kupfer, D. J. (1989). The Pittsburgh Sleep Quality Index: A new instrument for psychiatric practice and research. Psychiatry Research, 28, 193–213. 10.1016/0165-1781(89)90047-4 2748771

[phy214353-bib-0013] Cagnacci, A. , Soldani, R. , Laughlin, G. A. , & Yen, S. S. (1996). Modification of circadian body temperature rhythm during the luteal menstrual phase: Role of melatonin. Journal of Applied Physiology, 80, 25–29. 10.1152/jappl.1996.80.1.25 8847311

[phy214353-bib-0014] Cagnacci, A. , Soldani, R. , Romagnolo, C. , & Yen, S. S. (1994). Melatonin‐induced decrease of body temperature in women: A threshold event. Neuroendocrinology, 60, 549–552. 7845546 10.1159/000126794

[phy214353-bib-0015] Cagnacci, A. , Soldani, R. , & Yen, S. S. (1993). The effect of light on core body temperature is mediated by melatonin in women. Journal of Clinical Endocrinology and Metabolism, 76, 1036–1038. 10.1210/jc.76.4.1036 8473378

[phy214353-bib-0016] Cain, S. W. , Dennison, C. F. , Zeitzer, J. M. , Guzik, A. M. , Khalsa, S. B. S. , Santhi, N. , … Duffy, J. F. (2010). Sex differences in phase angle of entrainment and melatonin amplitude in humans. Journal of Biological Rhythms, 25, 288–296. 10.1177/0748730410374943 20679498 PMC3792014

[phy214353-bib-0017] Cajochen, C. , Münch, M. , Kobialka, S. , Kräuchi, K. , Steiner, R. , Oelhafen, P. , … Wirz‐Justice, A. (2005). High sensitivity of human melatonin, alertness, thermoregulation, and heart rate to short wavelength light. Journal of Clinical Endocrinology Metabolism, 90, 1311 10.1210/jc.2004-0957 15585546

[phy214353-bib-0018] Campbell, S. S. , & Broughton, R. J. (1994). Rapid decline in body temperature before sleep: Fluffing the physiological pillow? Chronobiology International, 11, 126–131. 10.3109/07420529409055899 8033241

[phy214353-bib-0019] Charkoudian, N. , & Stachenfeld, N. (2016). Sex hormone effects on autonomic mechanisms of thermoregulation in humans. Autonomic Neuroscience, 196, 75–80. 10.1016/j.autneu.2015.11.004 26674572

[phy214353-bib-0020] Charloudian, N. , & Stachenfeld, N. (2014). Reproductive hormone influences on thermoregulation in women. Comprehensive Physiology, 4, 793–804. 24715568 10.1002/cphy.c130029

[phy214353-bib-0021] de Mouzon, J. , Testart, J. , Lefevre, B. , Pouly, J. L. , & Frydman, R. (1984). Time relationships between basal body temperature and ovulation or plasma progestins. Fertility and Sterility, 41, 254–259. 10.1016/S0015-0282(16)47600-4 6421622

[phy214353-bib-0022] Diffey, B. , Piers, L. S. , Soares, M. J. , & O'Dea, K. (1997). The effect of oral contraceptive agents on the basal metabolic rate of young women. British Journal of Nutrition, 77, 853–862. 10.1079/BJN19970084 9227183

[phy214353-bib-0023] dos Reis, C. M. , de Melo, N. R. , Meirelles, E. S. , Vezozzo, D. P. , & Halpern, A. (2003). Body composition, visceral fat distribution and fat oxidation in postmenopausal women using oral or transdermal oestrogen. Maturitas., 46, 59–68. 10.1016/S0378-5122(03)00159-2 12963170

[phy214353-bib-0024] Driver, H. S. , Dijk, D. J. , Werth, E. , Biedermann, K. , & Borbély, A. A. (1996). Sleep and the sleep electroencephalogram across the menstrual cycle in young healthy women. Journal of Clinical Endocrinology Metabolism, 81, 728–735. 8636295 10.1210/jcem.81.2.8636295

[phy214353-bib-0025] Du Bois, E. F. (1921). The basal metabolism in fever. JAMA, 77, 352–355.

[phy214353-bib-0026] Dzaja, A. , Arber, S. , Hislop, J. , Kerkhofs, M. , Kopp, C. , Pollmächer, T. , … Porkka‐Heiskanen, T. (2005). Women's sleep in health and disease. Journal of Psychiatric Research, 39, 55–76. 10.1016/j.jpsychires.2004.05.008 15504424

[phy214353-bib-0027] Ferrannini, E. (1988). The theoretical bases of indirect calorimetry: A review. Metabolism, 37, 287–301. 10.1016/0026-0495(88)90110-2 3278194

[phy214353-bib-0028] Flatt, J. P. (1987). Dietary fat, carbohydrate balance, and weight maintenance: Effects of exercise. American Journal of Clinical Nutrition, 45, 296–306. 10.1093/ajcn/45.1.296 3799520

[phy214353-bib-0029] Frascarolo, P. , Schutz, Y. , & Jéquier, E. (1990). Decreased thermal conductance during the luteal phase of the menstrual cycle in women. Journal of Applied Physiology, 69, 2029–2033. 10.1152/jappl.1990.69.6.2029 2076997

[phy214353-bib-0030] Hessemer, V. , & Bruck, K. I. (1985). Influence of menstrual cycle on shivering, skin blood flow, and sweating responses measured at night. Journal of Applied Physiology, 59, 1902–1910. 10.1152/jappl.1985.59.6.1902 4077797

[phy214353-bib-0031] Hochachka, P. W. , & Somero, G. N. (1984). Biochemical Adaptation. Princeton: Princeton University Press.

[phy214353-bib-0032] Horne, J. A. , & Ostberg, O. (1976). A self‐assessment questionnaire to determine morningness‐ eveningness in human circadian rhythms. International Journal of Chronobiology, 4, 97–110. 1027738

[phy214353-bib-0033] Horvath, S. M. , & Drinkwater, B. L. (1982). Thermoregulation and the menstrual cycle. Aviation, Space and Environmental Medicine, 53, 790–794. 7181811

[phy214353-bib-0034] Howe, J. C. , Rumpler, W. V. , & Seale, J. L. (1993). Energy expenditure by indirect calorimetry in premenopausal women: Variation within one menstrual cycle. Journal of Nutritional Biochemistry, 4, 268–273. 10.1016/0955-2863(93)90096-F

[phy214353-bib-0035] Kayaba, M. , Park, I. , Iwayama, K. , Seya, Y. , Ogata, H. , Yajima, K. , … Tokuyama, K. (2017). Energy metabolism differs between sleep stages and begins to increase prior to awakening. Metabolism, 69, 14–23. 10.1016/j.metabol.2016.12.016 28285643

[phy214353-bib-0036] Kendrick, Z. V. , Steffen, C. A. , Rumsey, W. L. , & Goldberg, D. I. (1987). Effect of estradiol on tissue glycogen metabolism in exercised oophorectomized rats. Journal of Applied Physiology, 63, 492–496. 10.1152/jappl.1987.63.2.492 3654408

[phy214353-bib-0037] Kräuchi, K. , Cajochen, C. , Möri, D. , Hetsch, C. , & Wirz‐Justice, A. (1997). Melatonin and S‐20098 advance circadian phase and nocturnal regulation of core body temperature. American Journal of Physiology, 272, R1178–R1188. 9140018 10.1152/ajpregu.1997.272.4.R1178

[phy214353-bib-0038] Kräuchi, K. , Cajochen, C. , Werth, E. , & Wirz‐Justice, A. (1999). Warm feet promote the rapid onset of sleep. Nature, 401, 36–37. 10.1038/43366 10485703

[phy214353-bib-0039] Kräuchi, K. , Cajochen, C. , & Wirz‐Justice, A. (2005). Thermophysiologic aspects of the three‐process‐model of sleepiness regulation. Clinics in Sports Medicine, 24, 287–300. 10.1016/j.csm.2004.12.009 15892924

[phy214353-bib-0040] Kräuchi, K. , Konieczka, K. , Roescheisen‐Weich, C. , Gompper, B. , Hauenstein, D. , Schoetzau, A. , … Flammer, J. (2014). Diurnal and menstrual cycles in body temperature are regulated differently: A 28‐day ambulatory study in healthy women with thermal discomfort of cold extremities and controls. Chronobiology International, 31, 102–113. 10.3109/07420528.2013.829482 24131147

[phy214353-bib-0041] Kräuchi, K. , & Wirz‐Justice, A. (2001). Circadian clues to sleep onset mechanisms. Neuropsychopharmacology, 25, S92–S96. 10.1016/S0893-133X(01)00315-3 11682282

[phy214353-bib-0042] Lee, K. A. , McEnany, G. , & Zaffke, M. E. (2000). REM sleep and mood state in childbearing women: Sleepy or weepy? Sleep, 23, 877–885. 10.1093/sleep/23.7.1b 11083596

[phy214353-bib-0043] Lee, K. A. , Shaver, J. F. , Giblin, E. C. , & Woods, N. F. (1990). Sleep patterns related to menstrual cycle phase and premenstrual affective symptoms. Sleep, 13, 403–409. 2287852

[phy214353-bib-0044] Meijer, G. A. , Westerterp, K. R. , Saris, W. H. , & ten Hoor, F. (1992). Sleeping metabolic rate in relation to body composition and the menstrual cycle. American Journal of Clinical Nutrition, 55, 637–640. 10.1093/ajcn/55.3.637 1550036

[phy214353-bib-0045] Melanson, K. J. , Saltzman, E. , Russell, R. , & Roberts, S. B. (1996). Postabsorptive and postprandial energy expenditure and substrate oxidation do not change during the menstrual cycle in young women. Journal of Nutrition, 126, 2531–2538. 10.1093/jn/126.10.2531 8857514

[phy214353-bib-0046] Moline, M. L. , Broch, L. , Zak, R. , & Gross, V. (2003). Sleep in women across the life cycle from adulthood through menopause. Sleep Medicine Reviews, 7, 155–177. 10.1053/smrv.2001.0228 12628216

[phy214353-bib-0047] Park, I. , Ochiai, R. , Ogata, H. , Kayaba, M. , Hari, S. , Hibi, M. , … Tokuyama, K. (2017). Effects of subacute ingestion of chlorogenic acids on sleep architecture and energy metabolism through activity of the autonomic nervous system: A randomised, placebo‐controlled, double‐blinded crossover trial. British Journal of Nutrition, 117, 979–984. 10.1017/S0007114517000587 28412986

[phy214353-bib-0048] Parry, B. L. , Mostofi, N. , LeVeau, B. , Nahum, H. C. , Golshan, S. , Laughlin, G. A. , & Gillin, J. C. (1999). Sleep EEG studies during early and late partial sleep deprivation in premenstrual dysphoric disorder and normal control subjects. Psychiatry Research, 85, 127–143. 10.1016/S0165-1781(98)00128-0 10220004

[phy214353-bib-0049] Quarta, C. , Mazza, R. , Pasquali, R. , & Pagotto, U. (2012). Role of sex hormones in modulation of brown adipose tissue activity. Journal of Molecular Endocrinology, 49, R1–R7. 10.1530/JME-12-0043 22460126

[phy214353-bib-0050] Rooney, T. P. , Kendrick, Z. V. , Carlson, J. , Ellis, G. S. , Matakevich, B. , Lorusso, S. M. , & McCall, J. A. (1993). Effect of estradiol on the temporal pattern of exercise‐induced tissue glycogen depletion in male rats. Journal of Applied Physiology, 75, 1502–1506. 10.1152/jappl.1993.75.4.1502 8282595

[phy214353-bib-0051] Rubinstein, E. H. , & Sessler, D. I. (1990). Skin‐surface temperature gradients correlate with fingertip blood flow in humans. Anesthesiology, 73, 541–545. 10.1097/00000542-199009000-00027 2393139

[phy214353-bib-0052] Santosa, S. , & Jensen, M. D. (2013). Adipocyte fatty acid storage factors enhance subcutaneous fat storage in postmenopausal women. Diabetes, 62, 775–782. 10.2337/db12-0912 23209188 PMC3581212

[phy214353-bib-0053] Schoffelen, P. F. M. , & Plasqui, G. (2018). Classical experiments in whole‐body metabolism: Open‐circuit respirometry—diluted ow chamber, hood, or facemask systems. European Journal of Applied Physiology, 118, 33–49. 29080000 10.1007/s00421-017-3735-5 PMC5754424

[phy214353-bib-0054] Shechter, A. , Boudreau, P. , Varin, F. , Diane, B. , & Boivin, D. B. (2011). Predominance of distal skin temperature changes at sleep onset across menstrual and circadian phases. J Biol Rhythm, 26, 260–270. 10.1177/0748730411404677 21628553

[phy214353-bib-0055] Solomon, S. J. , Kurzer, M. S. , & Calloway, D. H. (1982). Menstrual cycle and basal metabolic rate in women. American Journal of Clinical Nutrition, 36, 611–616. 10.1093/ajcn/36.4.611 7124662

[phy214353-bib-0056] Stephenson, L. A. , & Kolka, M. A. (1985). Menstrual cycle phase and time of day alter reference signal controlling arm blood flow and sweating. American Journal of Physiology, 249, R186–R191. 10.1152/ajpregu.1985.249.2.R186 4025576

[phy214353-bib-0057] Steward, R. G. , Bateman, L. A. , Slentz, C. , Stanczyk, F. Z. , & Price, T. M. (2016). The impact of short‐term depot‐medroxyprogesterone acetate treatment on resting metabolic rate. Contraception, 93, 317–322. 10.1016/j.contraception.2016.01.001 26772904

[phy214353-bib-0058] Strassman, R. J. , Qualls, C. R. , Lisansky, E. J. , & Peake, G. T. (1991). Elevated rectal temperature produced by all‐night bright light is reversed by melatonin infusion in men. Journal of Applied Physiology, 71, 2178–2182. 10.1152/jappl.1991.71.6.2178 1778910

[phy214353-bib-0059] Stubbs, R. J. , Harbron, C. G. , Murgatroyd, P. R. , & Prentice, A. M. (1995). Covert manipulation of dietary fat and energy density: Effect on substrate flux and food intake in men eating ad libitum. American Journal of Clinical Nutrition, 62, 316–329. 10.1093/ajcn/62.2.316 7625338

[phy214353-bib-0060] Tokuyama, K. , Ogata, H. , Katayose, Y. , & Satoh, M. (2009). Algorithm for transient response of whole body indirect calorimeter: Deconvolution with a regularization parameter. Journal of Applied Physiology, 106, 640–650. 10.1152/japplphysiol.90718.2008 19008487

[phy214353-bib-0061] Webb, P. (1986). 24‐hour energy expenditure and the menstrual cycle. American Journal of Clinical Nutrition, 44, 614–619. 10.1093/ajcn/44.5.614 3766447

[phy214353-bib-0062] Zulley, J. , Wever, R. , & Aschoff, J. (1981). The dependence of onset and duration of sleep on the circadian rhythm of rectal temperature. Pflügers Arch, 391, 314–318. 10.1007/BF00581514 7312563

